# Detergent‐free isolation and characterization of amyloid precursor protein C99 in *E. coli* native lipid‐nanodiscs using non‐ionic polymer

**DOI:** 10.1002/pro.70276

**Published:** 2025-08-19

**Authors:** Gaurav Sharma, Bankala Krishnarjuna, Volodymyr M. Hiiuk, Magdalena I. Ivanova, Pavel Nagorny, Ayyalusamy Ramamoorthy

**Affiliations:** ^1^ Biophysics Program The University of Michigan Ann Arbor Michigan USA; ^2^ Department of Chemistry The University of Michigan Ann Arbor Michigan USA; ^3^ Biomedical Engineering, Michigan Institute for Neuroscience, Macromolecular Science and Engineering The University of Michigan Ann Arbor Michigan USA; ^4^ Department of Neurology The University of Michigan Ann Arbor Michigan USA; ^5^ Department of Chemical and Biomedical Engineering, FAMU‐FSU College of Engineering Florida State University Tallahassee Florida USA; ^6^ National High Magnetic Field Laboratory Florida State University Tallahassee Florida USA; ^7^ Institute of Molecular Biophysics Florida State University Tallahassee Florida USA; ^8^ Present address: T.N. SYS Meryl Pharma Ujjain India; ^9^ Present address: Department of Biochemistry All India Institute of Medical Sciences Guwahati India

**Keywords:** Alzheimer's disease, amyloid precursor protein (APP‐C99), C99 oligomer, detergent‐free purification, nanodisc, non‐ionic polymer

## Abstract

Alzheimer's disease (AD), a progressive neurodegenerative disorder, is characterized by cognitive decline resulting from neuronal cell death. A key contributor to AD pathology is C99, a membrane‐bound β‐secretase‐cleaved fragment of amyloid precursor protein (APP). C99 plays a central role in generating amyloid‐beta (Aβ) isomers, which are directly implicated in disease progression. Understanding its structure and lipid interactions is essential for elucidating its mechanistic role in AD and guiding therapeutic development. C99 has been studied in membrane mimetics such as micelles, bicelles, and reconstituted nanodiscs. Although reconstituted nanodiscs provide a native‐like lipid‐bilayer environment, the use of detergents prior to reconstitution has been reported to disrupt native folding and lipid‐protein interactions. In this study, we successfully isolated and purified C99 along with its associated lipids directly from *E. coli* cell membranes using a non‐ionic pentyl‐inulin polymer, avoiding the need for detergents. The purified C99‐containing pentyl‐inulin nanodiscs were characterized using SDS‐PAGE, Western blotting, dynamic light scattering (DLS), ^1^H NMR spectroscopy, matrix‐assisted laser desorption/ionization time‐of‐flight (MALDI‐TOF) mass spectrometry, and liquid chromatography–mass spectrometry (LC–MS). Notably, we observed SDS‐stable oligomers of C99. DLS and ^1^H NMR confirmed the presence of large particles composed of pentyl‐inulin and *E. coli* lipids. MALDI‐TOF and LC–MS verified the molecular mass and amino acid sequence of C99, respectively. We propose that this detergent‐free method for the direct isolation of C99 and native lipids using non‐ionic pentyl‐inulin may serve as a valuable tool for investigating the C99‐secretase complex and for developing compounds aimed at inhibiting the production of amyloid‐beta isomers.

## 1 INTRODUCTION

Membrane proteins constitute approximately 60% of the drug targets and 30% of the mammalian proteome (Arinaminpathy et al., 2009; Errey & Fiez‐Vandal, 2020; Levental & Lyman, 2023; Overington et al., 2006). Being gate‐keepers, membrane proteins are essential to carry out important cellular functions, including transmitting molecular information and transporting essential molecules across the cell membrane (Ma et al., 2024; Wu et al., 2023). Many devastating diseases are also caused by the functional impairment of membrane proteins (Wisedchaisri et al., 2023; Yu et al., 2023). Therefore, understanding their structure–function relationship holds much potential for drug design and development (Batool et al., 2019; Bou‐Assaf & Marshall, 2020; Yang et al., 2021).

While the importance of understanding the dynamic structure and function of membrane proteins is well recognized, significant challenges remain in achieving successful structural studies despite recent advances in cryo‐electron microscopy (cryoEM) and solid‐state NMR spectroscopy (Zhou et al., 2025). One of the primary obstacles to obtaining atomic‐resolution structural information lies in the reconstitution of membrane proteins into membrane mimetics that preserve their native fold and functional integrity, which is essential for biophysical characterization (Carpenter et al., 2008; Miles & Wallace, 2016; Opella, 2013; Tiefenauer & Demarche, 2012).

Traditionally, membrane proteins have been purified utilizing various detergents and then reconstituted into membrane mimetics for in vitro characterization (Bayburt et al., 2002; Bayburt & Sligar, 2010; Krishnarjuna & Ramamoorthy, 2022; Ritchie et al., 2009; Shen et al., 2013). Studies have reported that using detergents in the purification protocols disrupts the native lipid‐protein interactions as all the cell membrane lipids are washed out (Bao et al., 2013; Julien et al., 2021; Krishnarjuna & Ramamoorthy, 2022; Rawlings, 2016; Urner et al., 2022; Yang et al., 2014; Yeung & Stanley, 2010). The subsequent reconstitution into micelles, vesicles, or bicelles may not represent a native‐like environment for a membrane protein. This is because these membrane‐mimicking systems are less stable due to uncontrolled fusion (Stępień et al., 2023) or due to the presence of detergents and curvature (Krishnarjuna & Ramamoorthy, 2022).

Recent studies have demonstrated the benefits of using nanodiscs that contain flat lipid‐bilayered discs surrounded by protective amphipathic belts (Bayburt et al., 2002; Denisov & Sligar, 2016; Sligar & Denisov, 2021). Different types of nanodisc‐forming belts have been reported, which include membrane scaffold protein (MSP), short peptides, and synthetic polymers. Although these nanodiscs containing planar lipid bilayers are better suited, the commonly used approach exposes a membrane protein to detergents during purification, which is not desirable for stable and functional reconstitution of many types of membrane proteins. Contrarily, recently demonstrated detergent‐free membrane protein isolation methods, where the cell membranes are solubilized using lipid‐solubilizing synthetic polymers or peptides/proteins, do not remove native cell membrane lipids; thus, the native lipid‐protein interactions are retained to render high stability and function (Drulyte et al., 2023; Krishnarjuna, Im, et al., 2022; Krishnarjuna & Ramamoorthy, 2022; Krishnarjuna, Ravula, & Ramamoorthy, 2022; Krishnarjuna, Sharma, Hiiuk, et al., 2024; Krishnarjuna, Sharma, Im, et al., 2024).

Although several studies have reported the use of detergent‐free membrane protein isolation methods for functional and structural studies in native/near‐native membrane environments, it is still not straightforward as each membrane protein is unique and poses many difficulties, especially those with low expression levels and with self‐assembling properties (Krishnarjuna & Ramamoorthy, 2022). One such protein is the amyloid precursor protein‐C99 (APP‐C99 or C99), which is associated with AD (Beel et al., 2010; Koch et al., 2023; Lane et al., 2018; Shen & Kelleher, 2007). APP undergoes two sequential proteolytic cleavages by β‐ and γ‐secretases before forming amyloids‐β (Aβ) isomers (Abraham et al., 2023; Chen et al., 2017; Davra & Benzeroual, 2023; Koch et al., 2023). Firstly, APP is cleaved by β‐secretase to form C99, a 99‐residue C‐terminal protein (known as β‐CTF), which undergoes a second cleavage by γ‐secretase to form Aβ isomers. The accumulation of Aβ peptide aggregates has been linked to AD, a condition that is becoming increasingly prevalent with the aging population (Alzheimer's A, 2024; Lane et al., 2018; Zilioli et al., 2024). Moreover, some studies have directly implicated C99 in AD pathogenesis (Jordà‐Siquier et al., 2022; Lauritzen et al., 2019; Tompa, 2002), making it an important membrane protein for mechanistic and drug‐development studies (Takasugi et al., 2023) Previous studies have characterized C99 by reconstituting it in membrane‐mimicking environments that utilized detergent‐based methods to extract C99; hence, the native lipid‐protein interactions are likely to be lost (Krishnarjuna, Sharma, Hiiuk, et al., 2024; Motoki et al., 2012; Vetrivel et al., 2005). Therefore, it is important to study C99 with its associated native lipids to understand the role of native lipids on the structure and function of C99. A recent cryo‐EM study reported that γ‐secretase processes C99 through stepwise helix unwinding in a cholesterol‐ and phospholipid‐rich environment (Guo et al., 2024). While not directly focused on lipid interactions, the study emphasized the importance of membrane composition in C99 processing. Replica exchange molecular dynamics (REMD) simulations of the full‐length C99 in membranes of varying thickness revealed that the structural states of its N‐ and C‐terminal domains are strongly influenced by the surrounding lipid environment (Pantelopulos et al., 2018). Specifically, thinner membranes promote β‐strand formation at the N‐terminus, which may facilitate Aβ aggregation. In contrast, thicker membranes favor α‐helical conformations that stabilize interactions with γ‐secretase and cytoplasmic binding partners. These observations underscore the importance of preserving native lipid‐protein interactions when studying C99 structure and function.

Recent studies suggest that cholesterol's influence on Aβ production and AD pathogenesis is largely mediated by its ability to shape membrane domains and modulate protein trafficking (Pantelopulos et al., 2024). Simulations and biophysical studies further demonstrated that membrane properties, including bilayer thickness and lipid composition, modulate C99 dimer topology and conformational dynamics (Dominguez et al., 2016; Li et al., 2022; Viswanath et al., 2015). Notably, C99 itself can remodel its surrounding membrane environment (Pantelopulos et al., 2022), and recent evidence indicates that neuronal cholesterol levels regulate C99 oligomerization and its amyloidogenic potential (Mesa et al., 2024). These findings position C99 as both a sensor and effector of membrane lipid composition. Therefore, a method that preserves native lipid–protein interactions can serve as a powerful platform to study how cholesterol influences C99 monomer‐dimer equilibrium and its structure–function relationships in AD.

Detergents such as DDM and CHAPS have been shown to destabilize transmembrane domains, alter protein oligomerization (Bao et al., 2013; Julien et al., 2021), and disrupt native lipids that are essential for maintaining the conformational integrity of membrane proteins like C99 and their interaction with processing enzymes (Krishnarjuna, Sharma, Hiiuk, et al., 2024; Kumar et al., 2024). These disruptions can compromise the structural and functional fidelity of C99, potentially leading to incomplete or misleading mechanistic insights. This underscores the need for alternative purification strategies that preserve the native lipid environment.

This study introduces a new method for isolating C99 from bacterial cell membranes using a charge‐free polymer. This approach is also adaptable to brain tissue samples from AD patients, enabling the isolation of C99‐native lipid complexes. As such, the method has significant potential for elucidating the role of native lipids in regulating the structure and function of C99. To implement this strategy, C99‐rich cell membranes were solubilized using the pentyl‐inulin polymer, and C99‐enriched *E. coli* lipid nanodiscs were subsequently purified and characterized using biophysical techniques.

## 2 MATERIALS AND METHODS

### 2.1 Expression of recombinant C99 using IPTG and autoinduction

The plasmid with the C99 gene was kindly provided by Professor Charles R. Sanders (Vanderbilt University School of Medicine). The plasmid was chemically transformed into a C41 bacterial strain prior to colony selection on a carbenicillin agar plate. A single colony was inoculated in LB medium, and the cells were grown overnight at 200 rpm, 37°C. Pre‐culture was inoculated in ZYM‐5052 autoinduction medium (Bentley et al., 2020; Krishnarjuna, Sharma, Hiiuk, et al., 2024; Studier, 2005), supplemented with 1 mM MgSO_4_, 1× trace metal mix (#T1001, Teknova), 0.1× BME vitamin (#B6891, Sigma‐Aldrich), and 100 μg/mL carbenicillin at the ratio of 0.3:100. The cells were grown at 37°C until OD_600_ reached between 0.8 and 1.0. Then, the temperature was lowered to 16–18°C, and the culture was grown for an additional 16–20 h to overexpress the protein by autoinduction. The cells were harvested by centrifugation at 8000 rpm for 10 min, 4°C, and the pellets were stored at −80°C. In the case of IPTG induction, cells were induced with 1 mM IPTG and allowed to express at 16°C for 16 h and at 37°C for 5 h. A 0.8 OD_600_ sample (equal concentration) was loaded onto the gel to verify expression under both IPTG‐induced and autoinduction conditions. Negative control samples (cells without protein expression) were cultured to confirm that the observed protein signals were specific to the expressed construct and not due to background expression or contamination.

### 2.2 Cell lysis and preparation of C99‐enriched membranes

Two protease inhibitor tablets (#11836170001, cOmplete™, Mini, EDTA‐free protease inhibitor cocktail, Roche) were dissolved in a 50 mL resuspension buffer (75 mM Tris—pH 7.8, 300 mM NaCl, 0.2 mM EDTA), and the cell pellets (12 g) were resuspended in the resuspension buffer. Cell lysis with Lysozyme (New Brunswick, NJ, USA) (5 mg/1 g cell paste) and DNase (Sigma‐Aldrich, St. Louis, Missouri, USA) (1 mg/mL – dissolved in 5 mM MgCl_2_) was done on ice for 10 min before sonication was carried out using a 13 mm sonicator probe (Thomas Scientific, LLC, NJ) at 40% amplitude. The physical lysis was performed by sonicating the cells for 9 cycles, with a 20 s pulse and 1 min rest on ice between each cycle. The soluble components were removed by centrifugation for 35 min at 18,500 rpm in a JA20 rotor (Beckman) and the insoluble C99‐rich cell membranes (pellet) were collected and washed once with the 50 mL resuspension buffer, followed by a final wash with resuspension buffer without EDTA. All the steps to prepare C99‐rich cell membranes were performed on ice, and protease inhibitors were used throughout the membrane preparation steps.

### 2.3 Pentyl‐inulin polymer synthesis and analysis

The pentyl‐inulin polymer (also called INPEN) with a degree of substitution (DS) value of 30% was synthesized following a previously published protocol (Ravula & Ramamoorthy, 2021). The degree of substitution, which represents the average number of pentyl groups attached per fructose unit, was estimated using the method outlined in the same protocol.

1.0 g of inulin from chicory root (5.55 mmol of fructose, 1.0 equiv.) purchased from Sigma‐Aldrich (#I2255) with an average degree of polymerization (DP) of n ≈ 14 was added to 30 mL of *N,N‐*Dimethylacetamide (DMAc;#803,235) and stirred at 50°C until inulin was dissolved completely. The resulting reaction mixture was then cooled to room temperature, and 444 mg of sodium hydride (11.10 mmol, 2.0 equiv., 60% NaH in mineral oil) was added in three portions over 1 h. The resulting mixture was reheated to 50°C and stirred for 45 min before cooling down to room temperature again. Subsequently, 1.03 mL of pentyl bromide (8.33 mmol, 1.5 equiv.) was added, and the reaction mixture was stirred at room temperature for ~2–2.5 days. The progress of the reaction was monitored by ^1^H NMR spectroscopy, and the reaction was stopped once the desired degree of substitution (30%) was achieved. To quench the reaction, ice‐cold ethanol was added, followed by diethyl ether. The formed precipitate was separated by slow centrifugation (3000 rpm for 10 min) and then washed with diethyl ether and ethanol. The resulting crude pentyl‐inulin polymer was dissolved in water and subjected to dialysis using a Spectra Por dialysis tubing (MWCO: 500–1000 Da; #131096, Cole‐Parmer), followed by lyophilization to obtain a white powder. The polymer was characterized by ^1^H NMR spectroscopy (Figure S1). ^1^H NMR spectrum of pentyl‐inulin in D_2_O was recorded on a Varian Vnmrs 700 (700 MHz) spectrometer.

### 2.4 Membrane solubilization with pentyl‐inulin polymer

We first verified the presence of the C99 in the membranes. Briefly, the membranes that were prepared at a concentration of 25 mg/mL (by dissolving 25 mg of membranes in 1 mL of buffer) in solubilization buffer (10 mM Tris pH 7.4, 100 mM NaCl, and protease inhibitor) were added to the pentyl inulin polymer (prepared in solubilization buffer) at a 1:1 (w/w) membrane to polymer ratio. The membranes‐polymer mixture, prepared in a total volume of 1 mL, was then incubated on a rotor at 4°C overnight for solubilization. On the next day, the mixture was spun at 10,000 rpm for 45 min at 4°C, and the supernatant, representing the solubilized nanodiscs sample, was gently pipetted out before confirming it using sodium dodecyl‐sulfate polyacrylamide gel electrophoresis (SDS‐PAGE). Membranes without polymer were used as a negative control. The efficiency of the polymer in forming empty nanodiscs was also verified (Figures S2–S4).

For a large‐scale solubilization, 3.6 g of membranes were resuspended in 50 mL of solubilization buffer (10 mM Tris pH 7.4, 100 mM NaCl, and two protease inhibitor tablets). After resuspension, 1.8 g of pentyl‐inulin polymer (1:0.5 membrane‐to‐polymer ratio) was added to the membrane solution. The sample volume was then adjusted to 150 mL, and the mixture was incubated overnight at 4°C with gentle mixing.

### 2.5 SDS‐PAGE and Western blot

Protein detection in the solubilized fractions was accomplished using SDS‐PAGE on pre‐cast GenScript gradient gels (4–20%) (Piscataway, NJ). The samples were mixed in a 1:1 ratio with 2x dye (#1610737, Bio‐Rad) and heated for 4 min at 90°C before loading onto the gel. The gel electrophoresis was performed using a Bio‐Rad SDS‐PAGE gel casting system (Bio‐Rad Laboratories, Inc.; Hercules, CA, USA) at 100 V for 10 min at room temperature first and then at 150 V for 60 min at room temperature. The proteins were stained using a Coomassie stain (0.1% Coomassie Brilliant Blue, 30% ethanol, and 10% acetic acid) and destained using destaining buffer (30% methanol and 10% acetic acid) before capturing them on a Bio‐Rad ChemiDoc MP imaging system.

For the Western blot, the gel was run as explained above. During gel electrophoresis, a PVDF membrane (Immun‐Blot Bio‐Rad 1,620,177, 26 cm × 3.3 m, 0.2 μm) was soaked in 100% methanol for 30 min. The membrane was then soaked in the transfer buffer (0.25 M Tris, 1.92 M glycine, and 20% methanol) for 10 min. The proteins from the SDS‐PAGE gel were transferred onto the PVDF membrane at 4°C, 110 V for 1.5 h. The PVDF membrane was blocked for 1 h in blocking buffer (TBS‐0.02 M Tris, 0.15 M NaCl, 0.1% Tween 20, 10% milk) at room temperature. After blocking, the membrane was incubated with the primary antibody (6E10; Cat# 803004, Bio Legend, USA, prepared at a 1:500 dilution in TBST with 5% milk) overnight at 4°C with gentle shaking. The blot was then washed 3 times with 5% milk in TBST before incubating with the secondary antibody (goat anti‐mouse horseradish peroxidase; Thermo Fisher Scientific; Cat#32230, prepared at a dilution of 1:5000 in the same buffer as primary antibody). The membrane was washed 3 times as previously, and the protein bands were visualized by the GENESys imaging system in the presence of substrate Eco bright Femto HRP (# EBFH100, Innovative Solutions, USA).

### 2.6 Affinity purification of C99

All the purification steps were performed at 4°C. The overnight solubilized membranes (150 mL) were spun down at 18,000 rpm, 4°C for 30–50 min. The supernatant was carefully collected and filtered using a 1.2‐micron cellulose acetate filter (Findlay, OH).

Five milliliter of nickel‐chelated resin was equilibrated with His binding buffer A (30 mM Tris, pH 8.0, 100 mM NaCl) prior to the addition of the solubilized protein. The mixture was incubated for 2 h at room temperature with gentle rotation. The bound C99 was loaded on a chromatography column (Bio‐Rad #7372512) to separate the protein from the filtrate. The beads were washed with 10 column volumes (CV) of binding buffer to remove unbound polypeptides. C99 was eluted using a stepwise gradient of imidazole ranging from 20 to 300 mM. The collected fractions were analyzed by SDS‐PAGE; those containing C99 were pooled for further use.

### 2.7 Size‐exclusion chromatography (SEC)

The affinity‐purified C99‐containing nanodiscs were concentrated to 4.5 mL using an Amicon 10 kDa concentrator. The size‐exclusion column (HiLoad 16/600 Superdex® 200 pg, Cytiva, #28‐9893‐35) was washed with 2 CV of milli‐Q and equilibrated with 2 CV of size‐ex buffer (20 mM potassium phosphate pH 7.4, 100 mM NaCl) before injecting the sample into the column. Both the milli‐Q and the buffer were stored on ice during chromatography. The mobile phase was run at a 1 mL/min flow rate on an ӒKTA Purifier FPLC system (GE Healthcare, Chicago, IL). The milli‐Q and the buffer were degassed (Ultrasonic cleaner, Cole‐Parmer, Chicago) by filtering with a 0.2‐μ filter (Millipore Steritop Vaccum filter, Merck) before purification.

### 2.8 Matrix‐assisted laser desorption/ionization time‐of‐flight (MALDI‐TOF) mass spectrometry

Matrix‐assisted laser desorption/ionization time‐of‐flight (MALDI‐TOF) mass spectrometry measurements were performed using a Bruker Autoflex mass spectrometer instrument under linear positive ion mode to determine the mass of the C99. On a 384‐well MTP stainless‐steel plate, a sample (0.75 μL) was spotted with an equal volume of the matrices (2,5‐dihydroxybenzoic acid (DHB) or sinapinic acid (SA)). After the matrix was added to the sample, the mixture was thoroughly mixed and allowed to air dry. Prior to data acquisition, the mass spectrometer was calibrated using mid‐mass range protein standards: insulin (5734.52 Da) and myoglobin (16952.31 Da). Most of the samples were desalted with either C4 or C18 resin ZipTip 10 μL tips (Merck Millipore, USA) before preparing spots for MALDI‐TOF. The data was analyzed using Bruker flex Analysis software.

### 2.9 Liquid chromatography‐mass spectrometry (LC–MS)

To further verify the protein integrity and to support the MALDI‐TOF data, liquid chromatography–mass spectrometry (LC–MS) was performed.

#### 2.9.1 Sample preparation

The band from the destained SDS‐PAGE gel was cut and treated for disulfide bond reduction and alkylation with 10 mM DTT and 10 mM IAA in 50 mM ammonium acetate and 4 M urea. The sample was further diluted to 2 M urea with 50 mM ammonium bicarbonate, and the in‐gel trypsin digestion was allowed at 37°C overnight. Tryptic peptides were extracted with 80% acetonitrile before drying them using Speedvac. The sample was reconstituted in 0.1% formic acid for desalting using C18 pipette tips (Pierce C18 Pipette Tips, Thermo Scientific).

#### 2.9.2 Data collection

LC–MS analysis was performed on an Ultimate 3600 nan‐UPLC (Thermo Scientific) coupled to an Orbitrap Fusion Lumos Tribrid mass spectrometer (Thermo Scientific). The reconstituted samples (~5 μL) were separated on a C18 column (Acclaim PepMap 100, C18, 75 μm × 50 cm, 2 μm, Thermo Scientific) over a 90 min gradient of H_2_O with 0.1% formic acid (solvent A) and 80% acetonitrile in H_2_O with 0.1% formic acid (solvent B) at a flow rate of 300 nL/min (0–5 min, 5% B; 5–70 min, 5%‐50% B; 70–80 min, 50%‐95 %B; 80–82 min, 95% B; 82–90 min, 5% B). The peptides were introduced into the mass spectrometer in positive ion mode on‐line via nano‐ESI. MS and tandem mass spectrometry (MS/MS) data were collected using a data‐dependent approach to perform high‐resolution peptide analysis. MS1 spectra were recorded within the m/z range of 375–1500 at a resolution of 120 K using the Orbitrap; the subsequent MS/MS spectra for peptide sequencing were generated by applying HCD (higher‐energy collisional dissociation in a multipole collision cell) at 32% normalized energy.

#### 2.9.3 Data analysis

MS and MS/MS spectra were searched for peptide and protein identification using Proteome Discoverer 2.2 (Thermo Scientific). Using the Sequest search algorithm, the search was conducted against protein sequences of *E. coli* (UniProt, reviewed, 2023.02). Mass tolerances of 5 ppm (MS1) and 0.02 Da (MS/MS) were applied. The database search was allowed for both static modifications, carbamidomethylation of cysteine (C) and dynamic modifications, including methionine oxidation (M), asparagine deamidation (N), and N‐terminal protein acetylation. Peptide and protein identifications were filtered at a 1% false discovery rate (FDR). To assess the reliability of the LC–MS/MS data, several key parameters were examined. The cross‐correlation score (XCorr), which reflects the quality of the match between experimental data and theoretical values; an XCorr value of 2 is generally considered indicative of a reliable identification. Additionally, a Sequest HT score above 100 is typically regarded as optimal for confident protein identification.

### 2.10 Dynamic light scattering (DLS)

The size distribution of C99 nanodiscs was measured on the Wyatt Technology DynaPro® NanoStar® DLS instrument. Five μicroliter of the sample purified using SEC was loaded in the quartz MicroCuvette (Wyatt Technology, CA), and the DLS measurements were recorded at 25°C. Ten acquisitions of 5 s each were taken and averaged to plot the size distribution profile.

### 2.11 NMR spectroscopy

The formation of nanodiscs and the interaction between C99 and nanodiscs were studied through 1D ^1^H NMR. The SEC‐purified protein (in 10 mM Kpi, 50 mM NaCl pH 7.4) was loaded in a 5 mm NMR tube, and the spectra were recorded at 298 K on a Bruker 500 MHz NMR spectrometer (Billerica, MA, USA) using a TXI‐probe. The NMR data were processed and analyzed using Bruker Topspin software (version 3.6.2).

## 3 RESULTS AND DISCUSSION

### 3.1 High C99 yields achieved using the autoinduction method

**FIGURE 1:**
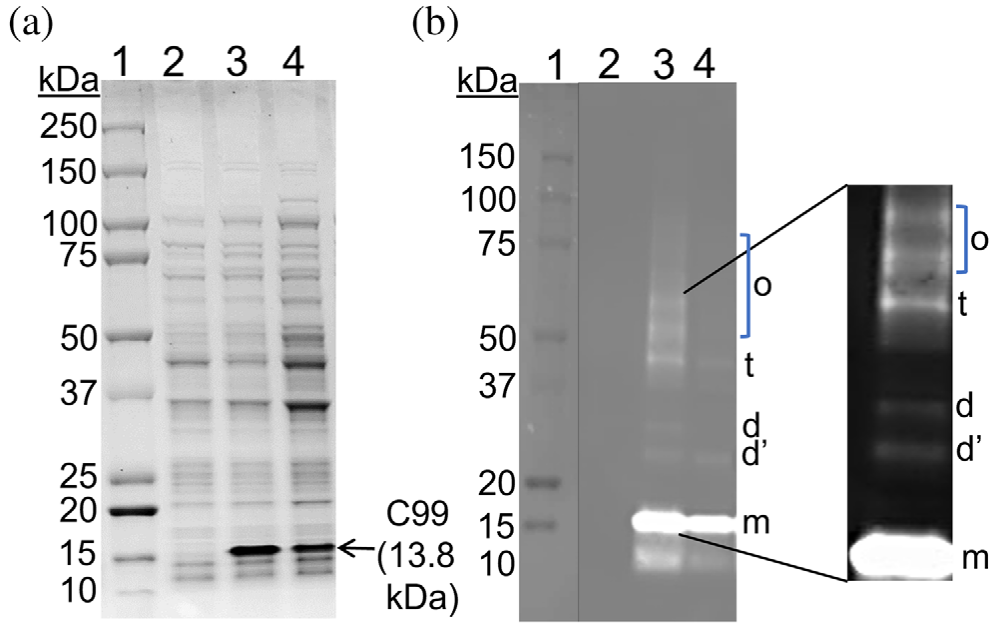
SDS‐PAGE/Western blot analysis of recombinant C99. a) C99 expression was analyzed by SDS‐PAGE. Lane 1: Protein marker; lane 2: Non‐induced negative control; lane 3: C99 overexpressed by the autoinduction method; and lane 4: C99 overexpressed by the IPTG‐induction method. b) Detection of C99 by Western blot; wells loaded with the same samples as in Figure 1a. m, Monomer; d, Dimer; d’, Degraded dimer; t, Trimer; and o, Oligomer. The dimer and oligomer bands in lane 3 are enlarged for clarity. Samples were loaded at a normalized concentration of 0.8 OD_600_ to assess protein expression levels. The protein band that appeared below the monomer is due to C99 degradation.

C99 was expressed in *E. coli* using autoinduction and IPTG induction methods (Figure 1a). The autoinduction was done at 16°C, whereas induction with IPTG was at 37°C. IPTG induction at a lower temperature (16°C) was also tested; however, it did not yield the optimal cell density required for the direct extraction method. Equal concentrations of total protein were loaded onto SDS‐PAGE gel to compare expression levels of C99 protein under different induction conditions. Autoinduction resulted in a markedly higher yield of C99 protein compared to IPTG induction, as evidenced by a more intense C99 protein band in the autoinduction sample (Figure 1a). Densitometric analysis indicated approximately a 1.5‐fold increase in the intensity of the autoinduction band. This result was corroborated by Western blot analysis (Figure 1b). The low protein yield in the IPTG‐induced samples is likely due to the predominant localization of the expressed protein in inclusion bodies rather than in the cell membrane (Figure S7). In contrast, autoinduction enhanced the localization of the protein in the membrane fraction, making it a more effective method for overexpressing C99.

Dimers, trimers, and higher‐order oligomers of C99 were also detected by Western blot (Figure 1b). These findings are consistent with previous studies reporting the presence of dimers, trimers, oligomers, and multimers of APP‐C99 (Dominguez et al., 2016; Fawzi et al., 2011; Gerber et al., 2017; Krishnarjuna, Sharma, Hiiuk, et al., 2024; Perrin et al., 2020). A recent study conducted on five membrane proteins proposed that oligomerization in membrane proteins is often driven by weak, transient interactions (Zhang et al., 2025; Zhang & MacKinnon, 2025). The trimer band appeared at an approximate size of 42 kDa (Figure 1b). The dimer band was expected to appear near 28 kDa; however, two bands were observed at a similar size. We believe that the band labeled with d corresponds to dimer and the band d' could be a degraded product of d.

Our observations under native‐like conditions are consistent with previous findings showing that membrane properties, including bilayer thickness, lipid composition, and cholesterol content, strongly influence C99 dimerization and conformational states (Beel et al., 2008; Dominguez et al., 2016; Pantelopulos et al., 2024; Viswanath et al., 2015). Notably, Pantelopulos et al. (2018) used REMD simulations to show that membrane thickness modulates C99 oligomerization by increasing flexibility in extramembrane regions and exposing interaction‐prone sites such as the G37G38 hinge. These simulations revealed multiple dimerization motifs, with varying helix crossing angles giving rise to distinct transmembrane dimer conformations whose stability is lipid dependent (Pantelopulos et al., 2018). More recent studies further showed that C99 can remodel its surrounding membrane, thereby altering its own oligomerization behavior and processing efficiency (Pantelopulos et al., 2022). Mesa et al. (2024) also reported that neuronal cholesterol levels modulate C99 oligomerization and amyloidogenic activity, emphasizing the importance of membrane composition in AD progression (Mesa et al., 2024).

Our detergent‐free extraction strategy preserves native lipid‐protein interactions and yields C99 in monomeric, dimeric, and higher‐order oligomeric forms. Unlike reconstitution‐based approaches, this method does not allow precise control over the lipid‐to‐protein ratio due to co‐extraction of endogenous lipids. However, downstream biophysical methods such as high‐resolution SEC and density gradient ultracentrifugation can be used to resolve and enrich specific oligomeric populations based on size and mass. Additionally, chemical cross‐linking under mild, native‐like conditions, followed by native PAGE or mass spectrometry, has been shown to stabilize transient C99 dimers (Hutchison et al., 2020). Site‐directed mutagenesis targeting key dimerization motifs (e.g., GXXXG, GXXXA) provides a complementary approach for modulating the oligomeric equilibrium (Pantelopulos et al., 2022). While the present study utilizes bacterial membranes, this strategy is extendable to cholesterol‐rich mammalian systems, including neurons and AD patient‐derived tissues.

### 3.2 Western blotting confirmed the C99 expression and its anomalous migration on SDS‐PAGE

We verified the C99 expression by Western blot before proceeding with the direct extraction approach (Figure 1b). The theoretical molecular weight of the C99 is 13.8 kDa; however, on SDS‐PAGE gel, the protein band was observed slightly above 15 kDa (Figure 1) as we reported earlier (Krishnarjuna, Sharma, Hiiuk, et al., 2024). The apparent increase in molecular weight of C99 observed on SDS‐PAGE is attributed to its anomalous migration, commonly referred to as “gel shifting”. Previous studies have documented the atypical migration behavior of transmembrane domains in varying concentrations of SDS (Emmanuel et al., 2025; Krishnarjuna et al., 2016; Lee et al., 2013; MacRaild et al., 2015; Morales et al., 2015;Rath et al., 2009; Walkenhorst et al., 2009). Rath et al. and others have proposed that gel shifting arises from altered detergent binding and differential solvation by SDS, which disrupt protein–protein interactions and favor protein‐detergent complexes (Rath et al., 2009; Walkenhorst et al., 2009). The authenticity of the C99 protein was confirmed by MALDI‐TOF and LC–MS/MS analyses, as detailed in Sections 3.7 and 3.8.

### 3.3 Small‐scale C99 pentyl‐inulin solubilization

**FIGURE 2:**
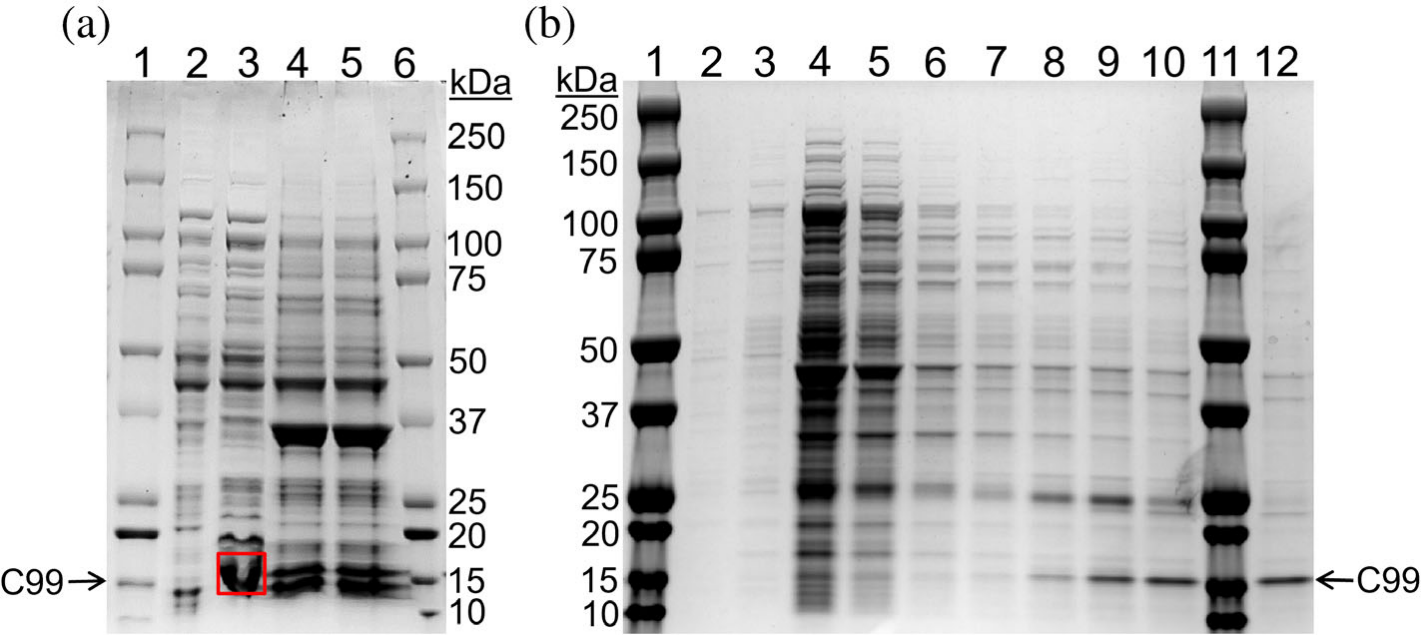
(a) SDS‐PAGE analysis of pentyl‐inulin‐solubilized *E. coli* cell membranes enriched with C99. Lanes 1 and 6 are protein markers; lane 2 (negative control) represents the supernatant of the membranes that were not treated with the polymer for solubilization; lane 3 contains the supernatant of C99 membranes that were solubilized with a pentyl‐inulin polymer (labeled with red box); lanes 4 and 5 represent pellets (insoluble fractions) from samples without and with polymer solubilization, respectively. (b) SDS‐PAGE analysis of detergent‐free extraction of C99 using Ni^2+^ affinity purification. Lanes 1 and 11 contain the protein molecular weight markers. Lanes 2–10 show eluted fractions at increasing imidazole concentrations of 20, 40, 50, 80, 120, 160, 200, 250, and 300 mM, respectively. Lane 12 corresponds to a second elution fraction using 300 mM imidazole.

Before directly extracting C99 at a large scale, a small‐scale solubilization experiment was performed using pentyl‐inulin polymer; this would prove the presence of C99 in the *E. coli* cell membranes prepared from the autoinduction system. The efficiency of the pentyl‐inulin polymer was evaluated by SEC analysis of nanodiscs assembled using a 7:3 (w/w) mixture of DMPC and DMPG lipids derived from commercial *E. coli* sources. The resulting SEC profile is shown in Figure S3. The pentyl‐inulin polymer was able to extract C99, as observed in lane 3 of Figure 2a. The cell membranes without the addition of polymer (representing negative control, lane 2) showed no band, confirming that the C99 band in lane 3 was due to pentyl‐inulin‐driven solubilization. C99 was also observed in the insoluble membrane fractions (in the insoluble pellet, lanes 4 and 5) due to incomplete solubilization of C99‐rich cell membranes.

### 3.4 Purification of C99 in bacterial nanodiscs using Ni‐NTA affinity chromatography

During an attempt to purify C99 using the Ni‐NTA chromatography column, we encountered difficulties due to the presence of large aggregates of crude cell membranes. Therefore, we opted to purify C99 using unpacked resin in a 50 mL centrifugation tube. The fractions eluted with an imidazole gradient were then analyzed using SDS‐PAGE (Figure 2b). Most unwanted proteins were eluted at imidazole concentrations ranging from 50 to 80 mM. Subsequently, at concentrations up to 160 mM of imidazole the band intensity of C99 on the SDS‐PAGE gel was not substantial. However, we observed high‐intensity protein bands at imidazole concentrations above 200 mM, indicating efficient binding of C99 to the resin. The purification profile obtained from the detergent‐free isolation method in this study is comparable to that attained through the detergent‐based method (Krishnarjuna, Sharma, Hiiuk, et al., 2024), suggesting excellent compatibility of pentyl‐inulin with metal‐chelated resins (Krishnarjuna & Ramamoorthy, 2022; Krishnarjuna, Ravula, & Ramamoorthy, 2022). The highest purity was achieved in the 300 mM imidazole elution fraction (second elution with 300 mM imidazole, lane 12), which was used for all subsequent experiments.

### 3.5 Purification of C99 in bacterial nanodiscs using SEC

**FIGURE 3:**
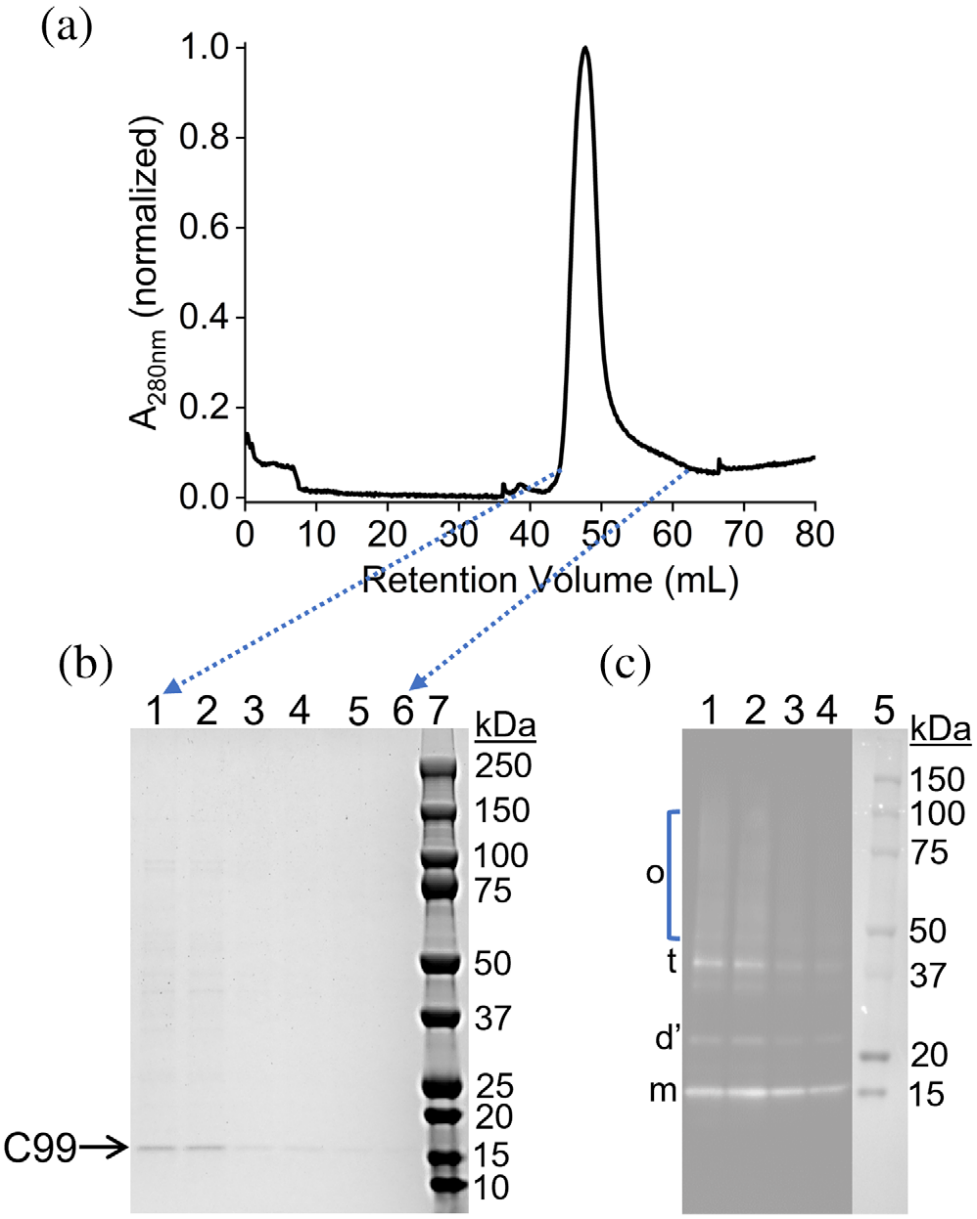
(a) SEC elution profile of directly extracted C99 nanodiscs, performed on the nickel‐purified fractions. The peak represents C99‐rich pentyl‐inulin nanodiscs. (b) SDS‐PAGE analysis on the SEC fractions collected between volumes 42 to 62 mL. The fractions loaded on the gel are shown with blue arrows. c) Western blot analysis on the first four fractions (Arinaminpathy et al., 2009; Errey & Fiez‐Vandal, 2020; Levental & Lyman, 2023; Overington et al., 2006) of SDS‐PAGE. m, monomers; d’, dimers (most likely a dimer); t, trimers; and o, oligomers.

SEC was performed to further purify the C99‐containing polymer nanodiscs (Figure 3a). SEC showed a single, mostly symmetric peak representing pentyl‐inulin‐C99 nanodiscs. The position of the peak in the SEC chromatogram resembled the peak position that was obtained through the reconstitution method (Krishnarjuna, Sharma, Hiiuk, et al., 2024), suggesting similar sizes of C99‐rich pentyl‐inulin nanodiscs. SDS‐PAGE analysis of the SEC fractions between volumes 42 mL to 62 mL indicated the protein bands corresponding to C99 (Figure 3b), and Western blotting confirmed the presence of C99 (Figure 3c). Western blot analysis revealed C99 bands corresponding to monomers (m), trimers (t), and higher‐order oligomers (o). Notably, a band above 20 kDa, likely representing a dimer (denoted as d’), was detected, although its precise position on the blot remains unclear. A band at a similar position was also observed in the overexpression samples (Figure 1b) Interestingly, the expected dimer band (d) is absent in Figure 3c, while only the putative degraded form (d’) is present. This may indicate that the dimer was degraded completely during or after purification, or alternatively, that it migrated differently due to distinct aggregation states. Intriguingly, oligomers are the predominant species in the detergent‐free isolated samples (Figures 1b and 3c), whereas higher‐order species (multimers) are more prominent in the detergent‐purified and nanodisc‐reconstituted C99 (Krishnarjuna, Sharma, Hiiuk, et al., 2024). However, low levels of oligomers were also detected in those later preparations. These differences highlight the uniqueness of the detergent‐free membrane protein isolation method over detergent‐based methods. Although challenging, further investigations to isolate and characterize these oligomers and higher‐order structures could provide valuable insights into the mechanisms underlying C99 stability.

### 3.6 Characterization of C99‐containing nanodiscs by DLS and ^1^H NMR

**FIGURE 4:**
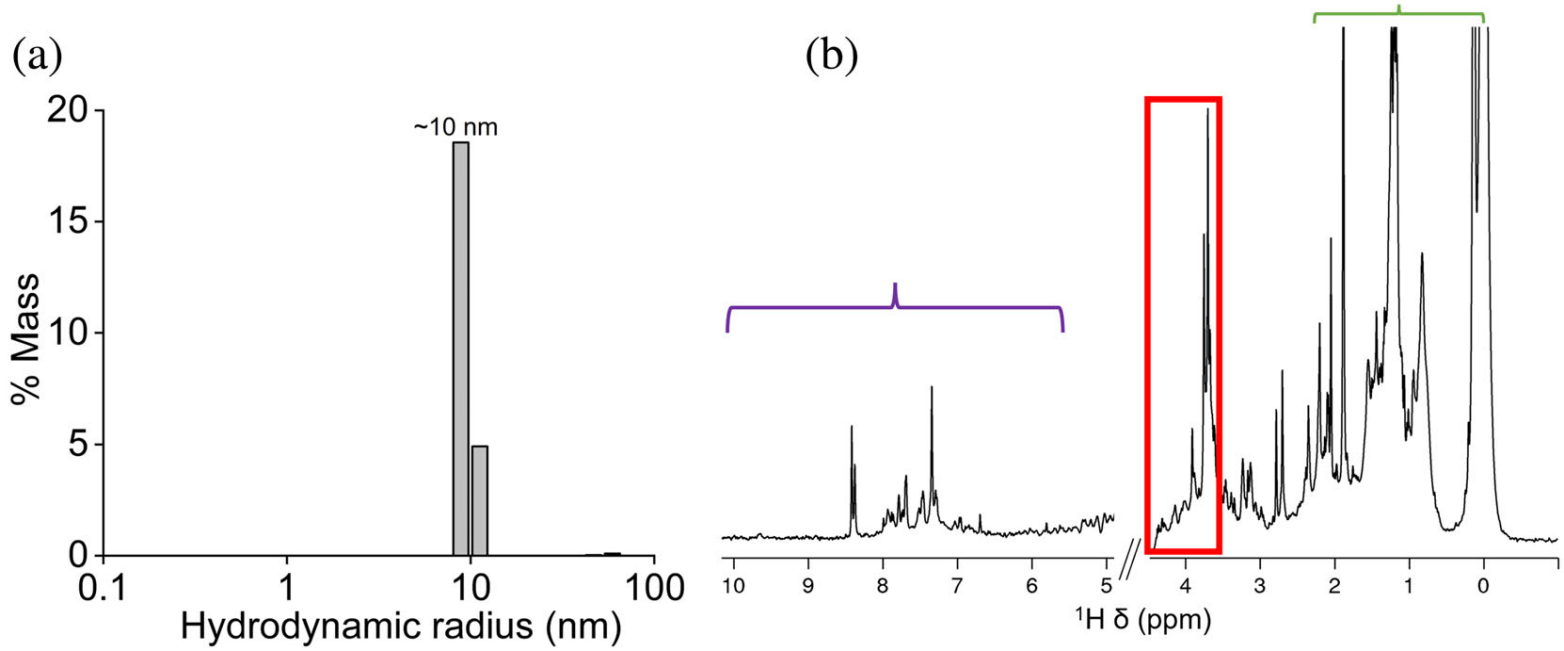
DLS and NMR analysis of C99 in *E. coli* lipid‐pentyl‐inulin nanodiscs. (a) DLS profile showing a hydrodynamic radius of ~10 nm. (b) ^1^H NMR spectrum of C99‐rich pentyl‐inulin nanodiscs. The amide region (violet bar), *E. coli* lipids and the pentyl‐inulin polymer (highlighted by red box and green bar).

The C99‐rich nanodiscs were analyzed using DLS measurements. The major nanodisc species, with a hydrodynamic radius of 10 ± 1 nm, were observed (Figure 4a). Again, the DLS results are in agreement with our previous study (Krishnarjuna, Sharma, Hiiuk, et al., 2024).

The composition of the SEC‐purified sample was analyzed by ^1^H NMR. The peaks representing polymer (from pentyl group and carbohydrate moiety) and bacterial lipids were observed (Figure 4b). However, the peaks corresponding to the C99 protein were not prominent due to line broadening from its interaction with lipids in nanometer‐sized polymer nanodiscs. To confirm the presence of C99, additional verification was performed using mass spectrometry.

### 3.7 MALDI analysis of C99‐nanodiscs

**FIGURE 5:**
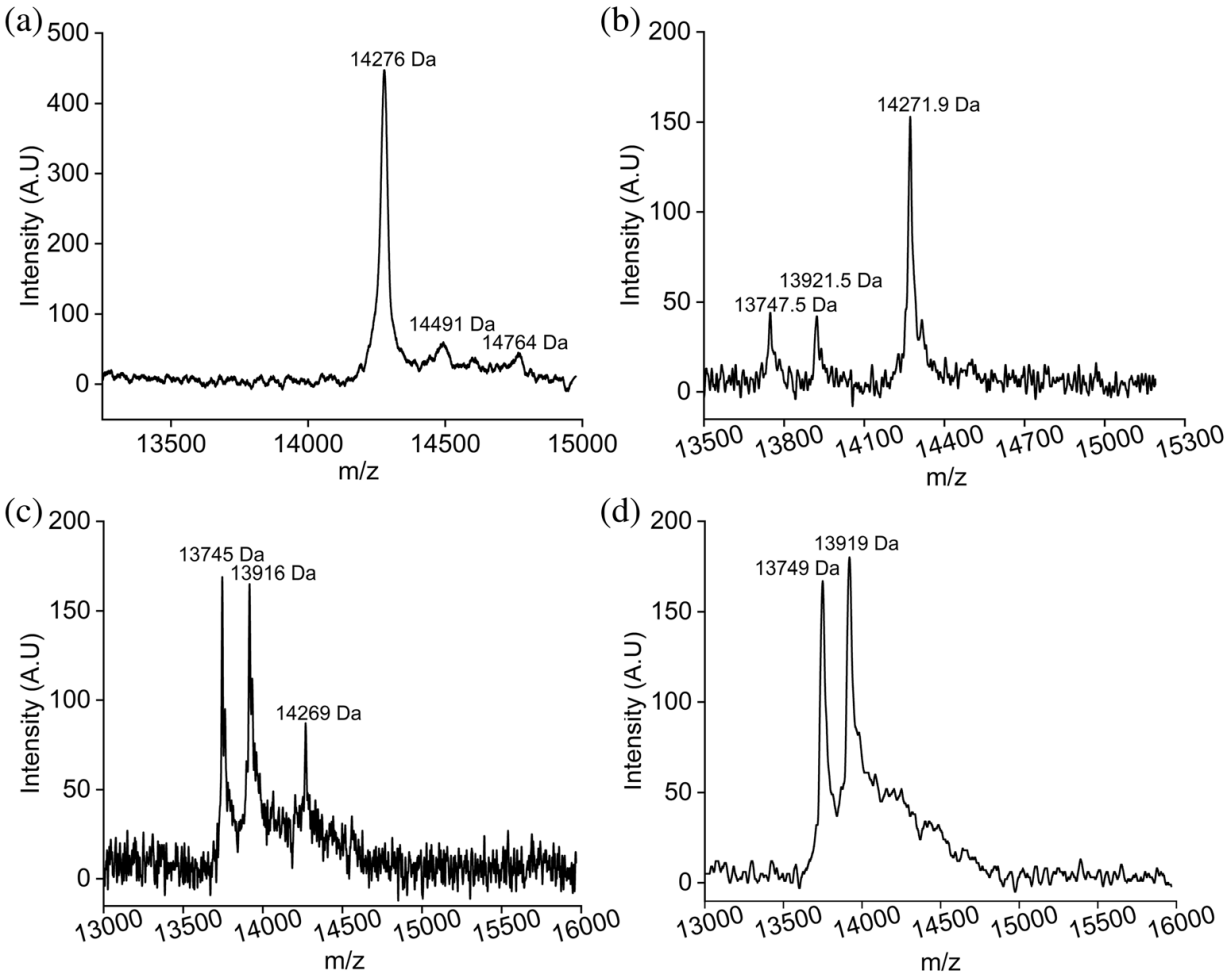
MALDI‐TOF MS spectra of C99 nanodiscs. (a). A peak corresponding to a molecular weight of 14,276 Da was observed, along with weak‐intensity peaks at higher molecular weights. The matrix used was SA. (b) After treatment with 3% Empigen, new peaks at molecular weights of 13,747.5 Da and 13,921.5 Da emerged. The matrix used was SA. (c) Following 3% Empigen treatment and analysis with the DHB matrix, the new peaks observed in Figure 5b showed increased intensity in Figure 5c, indicating improved resolution with the DHB matrix. (d) After treating C99 nanodiscs with 3% Empigen and 6 M GdnHCl (1:1) treatment, a significant suppression in the 14,269 Da peak was observed. The samples were desalted with C4 or C18 resin ZipTip 10 μL tips (Merck Millipore, USA) and mixed with SA, DHB, or CHCA matrices in a 1:1 ratio before spots on 384‐well stainless‐steel targets were prepared. Ionization was performed on a Bruker Autoflex mass spectrometer, and data were analyzed using Bruker flexAnalysis software.

The molecular mass of C99 in bacterial native nanodiscs was verified utilizing MALDI‐TOF. The expected mass of C99 is 13,779.76 Da; however, the mass values obtained through MALDI were 14,276 Da, 14,491 Da, and 14,764 Da (Figure 5a), which are higher than expected. The observed increase in molecular weight (~500 to 1000 Da) may result from the association of one or more bacterial lipids with the C99 protein, particularly since C99 was isolated from *E. coli* native lipid membranes without denaturation during extraction. While strong interactions with other biomolecules or self‐aggregation cannot be ruled out, these possibilities are beyond the scope of this study. To investigate and confirm the mass difference indicated by the C99 peaks, the samples were treated with the detergent Empigen, both in the presence and absence of denaturants such as guanidine hydrochloride (GdnHCl) and urea.

Two news peaks at 13,747.5 Da and 13,921.5 Da (in the near‐expected region), along with a peak at 14,271.9 Da (with a decrease in intensity by 3‐fold) were observed (Figure 5b). The intensity‐specific correlation indicates a possible interaction between lipids and C99 or C99 self‐aggregation. Upon changing the matrix to DHB while keeping all the other parameters the same, as kept for Figure 5b, high‐intensity peaks in the near‐expected area emerged (Figure 5c). The peaks at 13,745 and 13,916 Da were higher (~3.8‐ and 3.9‐fold, respectively) in intensity than what was obtained with SA. The peak at 14,269 went further down to an intensity of 87, inferring a 1.75‐fold decrease when compared with Figure 5b. Compared to Figure 5a, a drastic decrement of 5‐fold was observed in the ~14,200 Da peak.

To completely remove the possible interactions, C99 was treated with 6 M GdnHCl (1:1) and 8 M Urea (1:1) before analyzing by MALDI‐TOF (Figure 5d and S5B). Interestingly, the 14,269 peak almost disappeared, and the intensity of the 14,269 peak significantly went down to 44 (Figure 5d) from 87 (Figure 5c), decreasing by two‐fold. Compared to Figure 5a, the fold difference in Figure 5d is highly significant (10‐fold). The urea‐treated C99 (Figure S5B, red peaks) was not significantly different from the Empigen only sample (Figure 5c); however, the 6 M GdnHCl sample was clearly better than 8 M Urea in the case of C99. Interestingly, when the samples were analyzed without desalting them, no signals were observed in MALDI‐TOF (Figure S5).

The presence of two peaks (13,749 and ~13,919 Da) suggests a heterogeneous nature of the sample. Using the reconstitution method, we recently reported the C99 size as 13,741 Da (Krishnarjuna, Sharma, Hiiuk, et al., 2024). In that study, both detergent and denaturant were used before reconstituting C99 into nanodiscs. Hence, the molecular mass of C99 would be more accurate as only one prominent species was detected. In the current study, the observed molecular mass of C99 was ~13,745 and ~13,916 Da (Figure 5c). The first species closely matches the reconstitution study, and the difference of a few Da could be due to the addition of adduct H^+^ ions or the intrinsic sensitivity of the instrument used. However, the second species (~13,916 Da, approximately 171 Da larger) could not be conclusively characterized, even after treating the sample with both detergents and denaturants. Future studies could explore alternative combinations of solubilizing agents to isolate a single homogeneous species. Promising conditions may include 6 M GdnHCl in combination with DTT, CHAPS, or SDS, as well as SDS treatment coupled with heat and reducing agents such as DTT. Additionally, biochemical assays could help elucidate the identity of the higher‐mass species; for example, deglycosylation using PNGase F or Endo H, or phosphatase treatment may reveal post‐translational modifications. The structural and functional relevance of this second species could also be investigated using biophysical approaches, such as circular dichroism (CD) spectroscopy or NMR, particularly following detergent or denaturant treatment. Binding assays with lipids, APP‐processing proteins, or conformation‐specific antibodies may uncover differential interaction profiles between the two forms. However, such analyses would require the isolation of individual species, a technically challenging task that falls outside the scope of the current study.

To validate our observations and further check the presence of C99 in the tested sample for MALDI‐TOF, LC–MS experiments were performed.

### 3.8 C99 sequence analysis by tandem LC–MS/MS

To further validate the presence of C99 in our direct‐extraction sample, tandem mass spectrometry (LC–MS/MS) was performed. LC–MS/MS analysis demonstrated substantial sequence coverage (Figure S6B), providing strong evidence for the presence of C99. Peptides bearing carbamylation (C), deamidation (D), and oxidation (M) modifications were also identified (Figure S65B). An XCorr value of 6.8, together with an HT score of 140, indicates a good fit and strong confidence in the data. Additionally, the peptide‐spectrum match (PSM) count was 54, and detection of nine unique peptides, both within optimal range, further supports the reliability of the results. A detailed summary of these parameters is provided in Figure S6A.

Several high‐confidence MS/MS spectra corresponding to distinct tryptic peptides were acquired (Figure S6B). A particularly illustrative example is the tryptic peptide “QYTSIHHGVVEVDAAVTPEER,” corresponding to residues 57–77 of C99. The fragmentation evidence for this peptide is presented in Figure S6D, where an annotated tandem MS/MS spectrum displays both b and y ion series. This detailed fragmentation pattern supports confident peptide identification and sequence validation. A comprehensive tabulation of all observed b and y ions for this peptide is included in Figure S6C. Taken together, the tandem LC–MS/MS analysis, coupled with Western blot validation, provides robust confirmation of C99 presence in the directly isolated sample, demonstrating high analytical concordance.

## 4 CONCLUSION

BC99 has been implicated in the pathophysiology of Alzheimer's disease (Jordà‐Siquier et al., 2022; Lauritzen et al., 2019). Although C99 has been extensively studied in various membrane‐mimetic systems, its purification has traditionally relied on the use of detergents or denaturing agents, which disrupt protein–protein interactions (Yang et al., 2014) and compromise native lipid‐protein interactions essential for its functional conformation (Krishnarjuna & Ramamoorthy, 2022; Yang et al., 2014). In this study, we directly isolated C99 along with its associated lipids from *E. coli* using a non‐ionic pentyl‐inulin polymer, thereby preserving its near‐native lipid environment. The resulting C99‐rich nanodiscs were subsequently purified, maintaining the integrity of the lipid‐protein complex. The presence of isolated C99 was confirmed by SDS‐PAGE and Western blot, where SDS‐stable dimers and oligomers were observed. The formation of C99‐containing nanodiscs was further supported by DLS, which showed a hydrodynamic radius of ~10 nm. NMR spectroscopy analysis verified the presence of *E. coli* lipids and pentyl‐inulin, confirming nanodiscs formation.

MALDI‐TOF confirmed the expected molecular mass of C99, consistent with a previous C99 reconstitution study (Krishnarjuna, Sharma, Hiiuk, et al., 2024), and also revealed a new species at ~13,900 Da that remains in equilibrium with the main species (~13,745 Da). Finally, the amino acid sequence of the isolated C99 was validated by tandem LC–MS/MS analysis. We propose that this optimized detergent‐free isolation method could be broadly applicable for the isolation and study of other challenging membrane proteins, including amyloid precursor protein.

## AUTHOR CONTRIBUTIONS

**Gaurav Sharma:** Methodology; data curation; investigation; validation; formal analysis; visualization; writing – original draft; writing – review and editing. **Bankala Krishnarjuna:** Conceptualization; methodology; investigation; validation; formal analysis; visualization; data curation; writing – review and editing. **Volodymyr M. Hiiuk:** Investigation. **Magdalena I. Ivanova:** Supervision; investigation; writing – review and editing; validation; resources; formal analysis. **Pavel Nagorny:** Supervision; resources. **Ayyalusamy Ramamoorthy:** Conceptualization; methodology; investigation; supervision; funding acquisition; project administration; resources; writing – review and editing; data curation; formal analysis.

## Supporting information


**DATA S1.** Supporting Information.

## Data Availability

The data that support the findings of this study are available from the corresponding author upon reasonable request.
