# Supplementary material for: Detergent‐free isolation and characterization of amyloid precursor protein C99 in E. coli native lipid‐nanodiscs using non‐ionic polymer
Source: Protein Sci. 2025 Aug 19;34(9):e70276. doi: 10.1002/pro.70276 (PMC12363408; doi:10.1002/pro.70276)
Supplement: Supplementary file 1 — DATA S1. Supporting Information. [file PRO-34-e70276-s001.pdf]

Supplementary Information

Detergent-free isolation and characterization of amyloid precursor protein C99 in *E. coli* native lipid-nanodiscs using non-ionic polymer

Gaurav Sharma,[a,b,c=] Bankala Krishnarjuna,[a,b,c=] Volodymyr M Hiiuk,[a,b] Magdalena I Ivanova,[a,d] Pavel Nagorny[b] and Ayyalusamy Ramamoorthy[a,b,c,e,f,g]*

[a]*Biophysics Program, The University of Michigan, Ann Arbor, MI 48109, United States*

[b]*Department of Chemistry, The University of Michigan, Ann Arbor, Michigan 48109, United States*

[c]*Biomedical Engineering, Michigan Institute for Neuroscience, Macromolecular Science and Engineering, The University of Michigan, Ann Arbor, MI 48109, United States*

[d]*Department of Neurology, The University of Michigan, Ann Arbor, MI 48109, United States*

[e]Department of Chemical and Biomedical Engineering, FAMU-FSU College of Engineering, Florida State University, Tallahassee, FL 32310, United States

[f]National High Magnetic Field Laboratory, Florida State University, Tallahassee, FL 32310, United States

[g]Institute of Molecular Biophysics, 91 Chieftan Way, Florida State University, Tallahassee, FL 32304, United States

[=] equal contribution

***Corresponding Author:**
Ayyalusamy Ramamoorthy
Email: aramamoorthy@fsu.edu

Current address:

Gaurav Sharma, *T.N. SYS Meryl Pharma, Ujjain, India - 456010*

Bankala Krishnarjuna, *Department of Biochemistry, All India Institute of Medical Sciences, Guwahati, India - 781101*

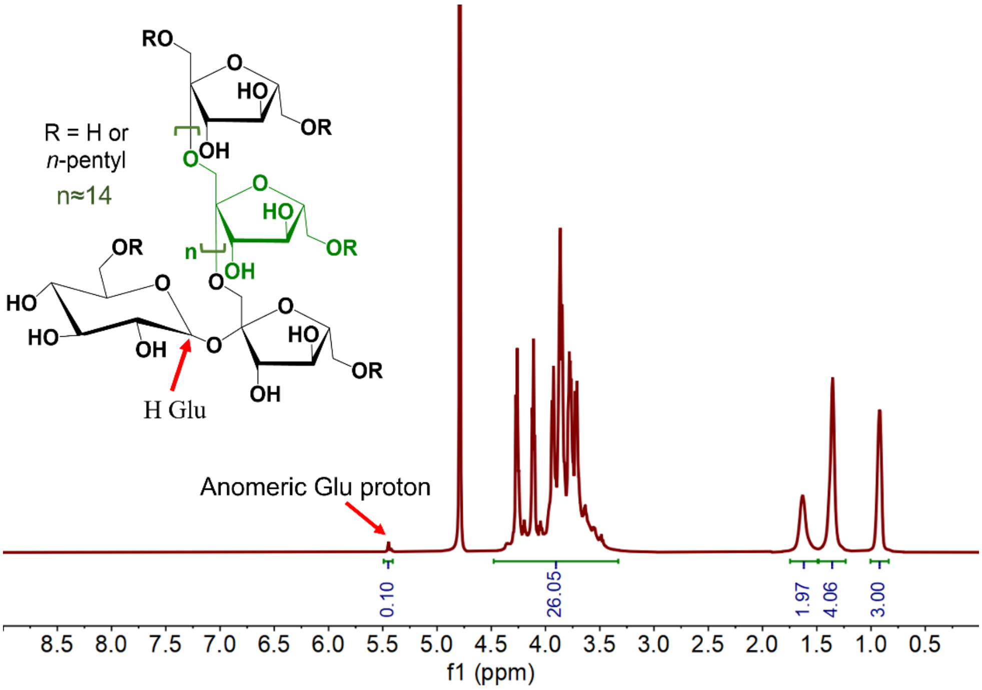

***Figure S1**. ^{1}H NMR spectrum of pentyl-inulin polymer with the degree of substitution of 30%. The spectrum was recorded on a Varian Vnmrs 700 (700 MHz) spectrometer using D_2O as a solvent. The spectrum was recorded at room temperature.*

Testing polymer efficiency to form nanodiscs

Polymer-nanodiscs were prepared by adding INPEN (100 mg/mL in 10 mM Tris buffer, pH 7.4, containing 100 mM NaCl) to DMPC/DMPG (7:3, w/w) in a 1:1 polymer/lipid ratio. After incubating the polymer/lipid mixture overnight at 4 °C with gentle mixing, a clear solution containing polymer-nanodiscs was obtained (**Fig. S2-C**). However, in the absence of INPEN polymer, the solution was turbid due to the formation of liposomes (**Fig. S2-A**). A phase transition from liposomes to nanodiscs was observed as soon as the polymer was added to the liposomes (**Fig. S2-B**).

Preparation of nanodiscs: 7 mg of DMPC and 3 mg of DMPG (both purchased from Avanti Polar Lipids, Alabaster, USA) were dissolved in a $CH_3OH/CHCl_3$ (1:1, v/v) separately. The solvents were evaporated using N_2 gas, and the residual solvent was completely removed under high vacuum overnight. The resulting dried lipid films were resuspended in 10 mM Tris buffer (pH 7.4) containing 100 mM NaCl. The lipids were completely solubilized with the help of 3-5 freeze-thaw cycles (using liquid nitrogen and hot water ~70°C). When resuspended, both the lipids were mixed together to form a total volume of 900 µL. The polymer was then added to the lipids in a 1:1 ratio to form nanodiscs (**Fig. S2-C**).

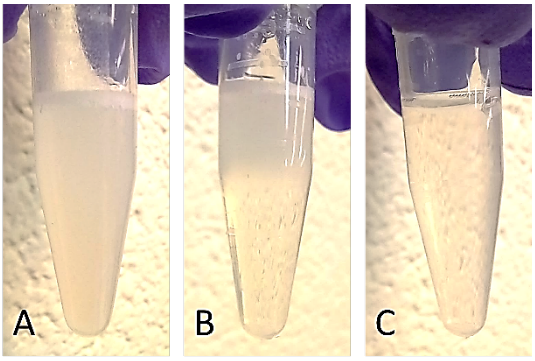

Figure S2. ***A****) DMPC/DMPG (7:3, w/w) liposomes; the sample was freeze-thawed 3-5 times.* ***B****) Solubilization of DMPC/DMPG lipids with INPEN polymer (1:1, w/w); the image was captured just after adding and mixing the polymer with lipids, and* ***C****) Clear solution indicating a stable polymer-nanodisc formation. The sample was gently rotated overnight at 4 °C. The images were taken at room temperature.*

The formed polymer-nanodiscs were purified by size-exclusion chromatography (SEC) using a GE ÄKTA Purifier FPLC System equipped with a SuperdexTM 200 Increase 10/300 GL column and a 214 nm detector. The experiment was performed at room temperature at a flow rate of 0.7 mL/min (**Fig. S3**).

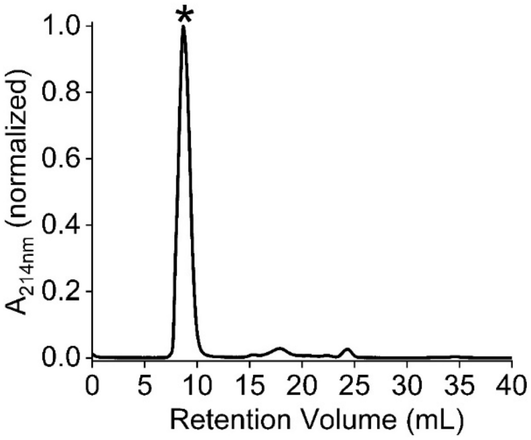

Figure S3. *SEC profile of polymer (INPEN) nanodiscs formed using commercial E. coli lipids (DMPC and DMPG at a ratio of 7:3 (w/w)). The higher peak (labeled with a star) at 8.65 mL represents nanodiscs.*

Dynamic light scattering was used to measure the hydrodynamic radius (R_{hyd}) of the SEC-purified empty nanodiscs. The DLS profile shows a monodisperse distribution with a hydrodynamic radius of ~10 nm, indicative of uniform nanodisc formation following purification (**Fig. S4**).

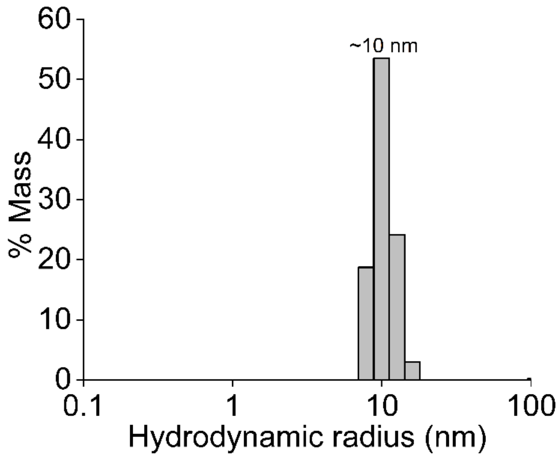

Figure S4. *DLS profile of SEC-purified empty nanodiscs showing a large complex with a hydrodynamic radius (R_{hyd}) of ~10 nm.*

Effect of detergents/denaturants on the MALDI-TOF outcome

Zip Tip desalting tips are generally used to improve the quality of the spectra by removing the salts, detergents, etc. Without the desalting step, the C99 was not observed (peaks in black) **(Fig. S5);** the spectra for non-desalted samples were observed in the baseline region. The clear peaks were observed after the desalting step (peaks in red). Urea-based denaturation did not render clean spectra; therefore, the baseline was also noisy **(Fig. S5-B)**.

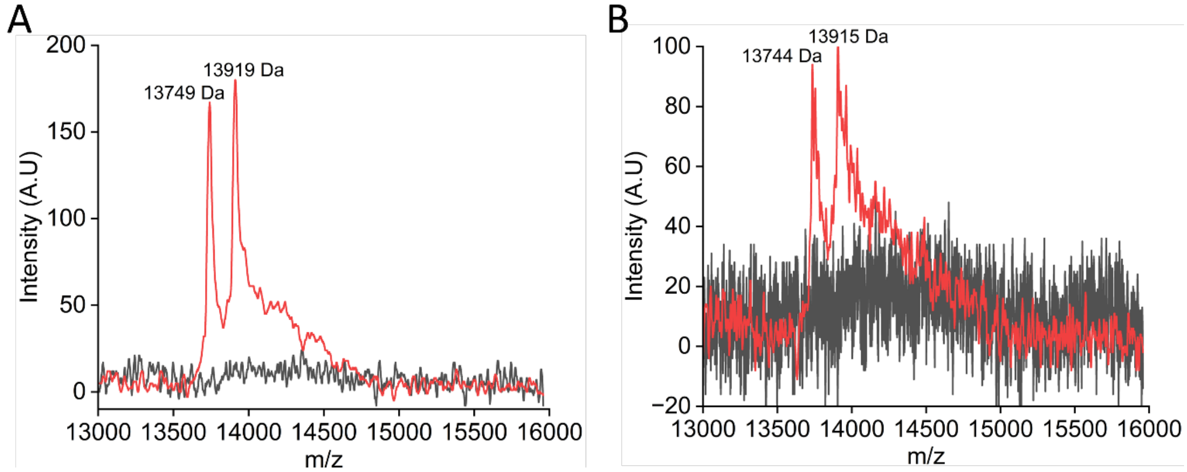

Figure S5. *MALDI-TOF MS spectra of C99 nanodiscs treated with* ***A)*** *3% Empigen detergent and 8M Urea (1:1).* ***B)*** *3% Empigen detergent and 6M GdnHCl (1:1). The samples shown in red were desalted using ZipTip, whereas the sample in black was spotted without the desalting step.*

C99 sequence analysis by tandem LC-MS/MS

LC-MS/MS tandem mass spectroscopy-derived data confirmed the presence of C99 in the direct-extraction *E. coli* fractions of C99. A summary of the parameters like Sequest HT scores, sequence coverage, PSMs, and unique peptides are tabulated in **Fig. S6-A**. The detailed

information about the C99 sequences covered (% coverage) and the modifications observed in the mass spectroscopy, with the confidence levels, are tabulated in **Fig. S6-B.**

The fragmentation evidence of one of the tryptic peptides "QYTSIHHGVVEVDAAVTPEER" (from 57 to 77 aa) is tabulated in **Fig. S6-C**; the spectra with b and y ions as depicted by annotated tandem MS-MS spectrum are shown in **Fig. S6-D.**

A

**Description**	**C99**
Score Sequest HT: Sequest HT	140
Coverage [%]	54
# PSMs	54
# Unique Peptides	9
MW [kDa]	13.8
Abundances (Grouped): Sample	221605198.7

B

Sequence	Modifications	Qvality PEP	# PSMs	Theo. MH+ [Da]	Abundances (Grouped)	Confidence
MDAEFRHDSGYEVHHQK	1xOxidation [M1]	0.000245934	3	2101.91446	682394.8516	High
MQQNGYENPTYK	1xDeamidated [N4]	0.00121451	1	1473.63147	3031083.25	High
MQQNGYENPTYK	1xOxidation [M1]	7.14958E-05	6	1488.64237	69237976	High
MQQNGYENPTYK	1xCarbamyl [N-Term]	0.000140621	2	1515.65327	1093814.875	High
MQQNGYENPTYKFFEQMQNQGR	1xDeamidated [N4]; 2xOxidation [M1; M17]	5.24367E-05	1	2771.18244	4015564	High
MQQNGYENPTYKFFEQMQNQGR	2xOxidation [M1; M17]	5.70633E-05	1	2770.19842	3696863.5	High
MQQNGYENPTYK	1xCarbamyl [N-Term]; 1xDeamidated [N4]	9.08499E-05	3	1516.63728	4270779.125	High
MQQNGYENPTYK	1xDeamidated [N4]; 1xOxidation [M1]	9.47731E-05	16	1489.62639	128841600.7	High
QYTSIHHGVVEVDAAVTPEER		0.00131546	3	2337.14696	2786620.586	High
FFEQMQNQGR	1xCarbamyl [N-Term]; 1xOxidation [M5]	0.00542983	2	1343.57971	780011.875	High
HLSKMQQNGYENPTYKFFEQMQNQGR	1xDeamidated [N]; 2xOxidation [M5; M21]	0.000675178	4	3236.45241	524872.7813	High
KQYTSIHHGVVEVDAAVTPEER		0.000160388	5	2465.24193	2298505.641	High
QYTSIHHGVVEVDAAVTPEERHLSK		0.0205805	1	2802.41693	27345.57227	High
KKQYTSIHHGVVEVDAAVTPEER		0.019737	4	2593.33689	317765.9297	High
MQQNGYENPTYKFFEQMQNQGR	2xDeamidated [N4; N8]; 1xOxidation [M17]	0.121478	1	2756.17154		High
MQQNGYENPTYK		0.152079	1	1472.64745		High

C

57 77
QYTSIHHGVVEVDAAVTPEER

#1	Immonium	b^{+}	b^{2+}	b^{3+}	Seq.	y^{+}	y^{2+}	y^{3+}	#2
**1**	101.07094	129.06585	65.03657	43.69347	**Q**				**21**
**2**	136.07569	292.12918	146.56823	98.04791	**Y**	2209.08839	1105.04783	737.03431	**20**
**3**	74.06004	393.17686	197.09207	131.73047	**T**	2046.02506	1023.51617	682.67987	**19**
**4**	60.04439	480.20889	240.60808	160.74115	**S**	1944.97738	972.99233	648.99731	**18**
**5**	86.09643	593.29295	297.15011	198.43584	**I**	1857.94535	929.47631	619.98663	**17**
**6**	110.07127	730.35187	365.67957	244.12214	**H**	1744.86129	872.93428	582.29195	**16**
**7**	110.07127	867.41078	434.20903	289.80844	**H**	1607.80237	804.40483	536.60564	**15**
**8**	30.03383	924.43224	462.71976	308.81560	**G**	1470.74346	735.87537	490.91934	**14**
**9**	72.08078	1023.50065	512.25397	341.83840	**V**	1413.72200	707.36464	471.91218	**13**
**10**	72.08078	1122.56907	561.78817	374.86121	**V**	1314.65359	657.83043	438.88938	**12**
**11**	102.05496	1251.61166	626.30947	417.87540	**E**	1215.58517	608.29622	405.86657	**11**
**12**	72.08078	1350.68008	675.84368	450.89821	**V**	1086.54258	543.77493	362.85238	**10**
**13**	88.03930	1465.70702	733.35715	489.24052	**D**	987.47416	494.24072	329.82957	**9**
**14**	44.04948	1536.74413	768.87570	512.91956	**A**	872.44722	436.72725	291.48726	**8**
**15**	44.04948	1607.78125	804.39426	536.59860	**A**	801.41011	401.20869	267.80822	**7**
**16**	72.08078	1706.84966	853.92847	569.62140	**V**	730.37299	365.69014	244.12918	**6**
**17**	74.06004	1807.89734	904.45231	603.30396	**T**	631.30458	316.15593	211.10638	**5**
**18**	70.06513	1904.95010	952.97869	635.65488	**P**	530.25690	265.63209	177.42382	**4**
**19**	102.05496	2033.99269	1017.49999	678.66908	**E**	433.20414	217.10571	145.07290	**3**
**20**	102.05496	2163.03529	1082.02128	721.68328	**E**	304.16155	152.58441	102.05870	**2**
**21**	129.11347				**R**	175.11895	88.06311	59.04450	**1**

Figure S6. A) *LC-MS/MS spectroscopy-driven data like Sequest HT scores, % sequence coverage, PSMs, and unique peptides are tabulated.* ***B)*** *The C99 sequences and the modifications that were observed in the mass spectroscopy are tabulated.* *The sequence-specific information is also mentioned.* ***C)*** *and* ***D)*** *depict b and y ions for sequence "QYTSIHHGVVEVDAAVTPEER" (from 57 to 77 aa), confirming the high confidence.*

D

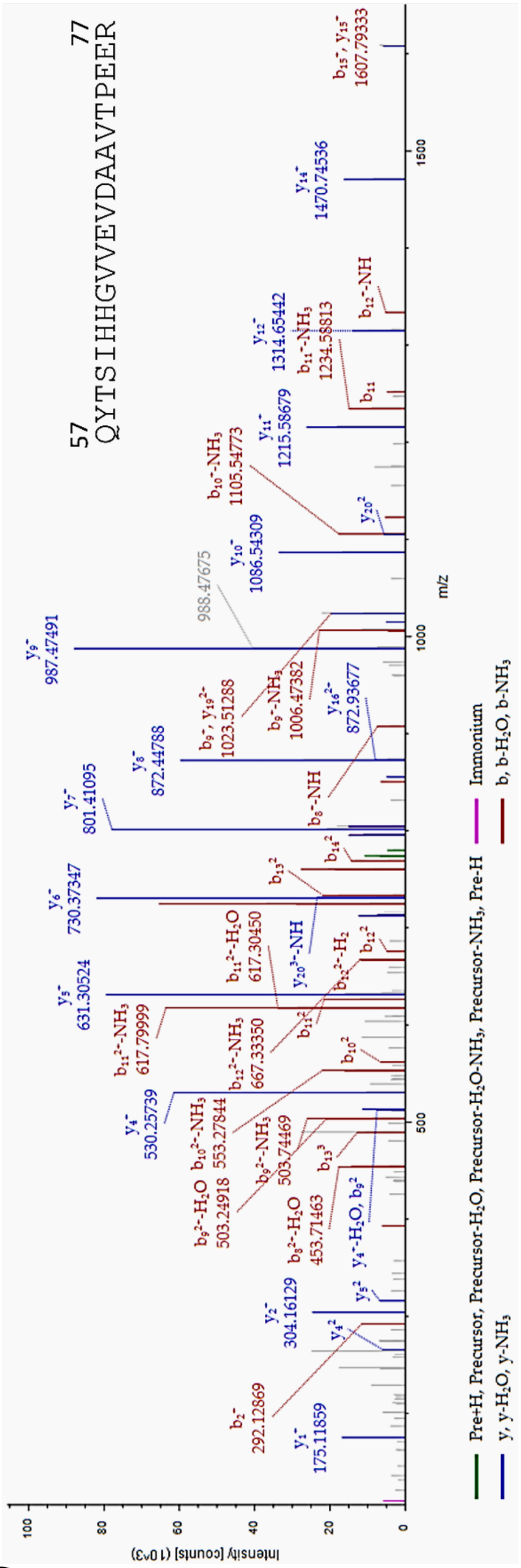

57 QYTSIHHGVVEVDAAVTPEER 77
Intensity [counts] (10^3)
m/z
y1+ 175.11859
b2+ 292.12869
y2+ 304.16129
y4^2
y5^2
y4+-H2O, b9^2
b8^2-H2O 453.71463
b13^3
b9^2-H2O 503.24918
b9^2-NH3 503.74469
b10^2-NH3 553.27844
y4+ 530.25739
b10^2
b12^2-NH3 667.33350
b11^2
b11^2-NH3 617.79999
b12^2-H2
y5+ 631.30524
b11^2-H2O 617.30450
y20^3-NH
b12^2
y6+ 730.37347
b13^2
b14^2
y7+ 801.41095
b8+-NH
y8+ 872.44788
b9+, y19^2+ 1023.51288
b9+-NH3 1006.47382
y16^2+ 872.93677
y9+ 987.47491
988.47675
y10+ 1086.54309
b10+-NH3 1105.54773
y20^2
y11+ 1215.58679
b11
b11+-NH3 1234.58813
y12+ 1314.65442
b12+-NH
y14+ 1470.74536
b15+, y15+ 1607.79333
Pre+H, Precursor, Precursor-H2O, Precursor-H2O-NH3, Precursor-NH3, Pre-H
Immonium
y, y-H2O, y-NH3
b, b-H2O, b-NH3

Localization of C99:

In several attempts, even at lower temperatures, we observed more C99 expression in inclusion bodies (lanes 7, 8, and 9) than in cell membranes (lanes 4, 5, and 6) when the IPTG induction method was used **(Fig. S7).**

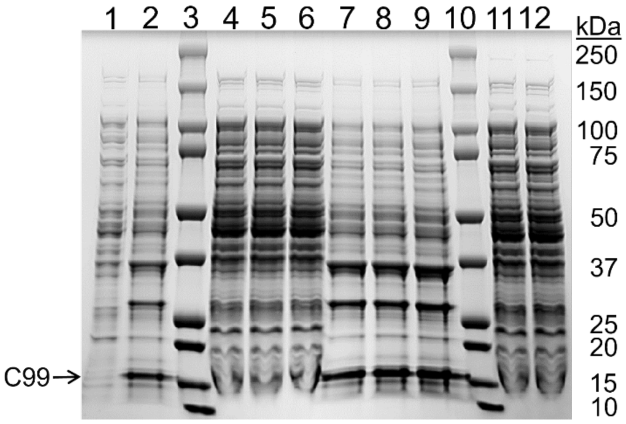

Figure S7. *SDS-PAGE analysis of C99 IPTG expression in E. coli and pentyl-inulin solubilized membranes. Lane 1: supernatant fraction after cell lysis of IPTG expressed cells (-ve control, no polymer added); lane 2: pellet fraction after cell lysis of IPTG expressed cells (containing membranes, no polymer, -ve control); lanes 4, 5 and 6: represent supernatant of O/N polymer solubilized C99 membranes (triplicates); lanes 7, 8 and 9: represents pellets fractions of 4,5 and 6 (which, polymer could not solubilize); lane 11 and 12: represents sample 4 and 5 without heating before loading onto the gel (to check if heating is obstructing the visibility of the bands). Lanes 3 and 10 represent protein markers.*
